# Corrosion Inhibition of Honeycomb Waste Extracts for 304 Stainless Steel in Sulfuric Acid Solution

**DOI:** 10.3390/ma12132120

**Published:** 2019-07-01

**Authors:** Femiana Gapsari, Kartika A. Madurani, Firman Mangasa Simanjuntak, Andoko Andoko, Hastono Wijaya, Fredy Kurniawan

**Affiliations:** 1 Laboratory of Metrology Industry, Department of Mechanical Engineering, Faculty of Engineering, Brawijaya University, Malang 65145, Indonesia; 2 Laboratory of Instrumentation and Analytical Sciences, Department of Chemistry, Faculty of Sciences, Institut Teknologi Sepuluh Nopember, Surabaya 60111, Indonesia; 3 World Premier Institute (WPI)-Advanced Institute for Materials Research, Tohoku University, Sendai 980-8577, Japan; 4 Mechanical Engineering Department, Faculty of Engineering, State University of Malang, Malang 65145, Indonesia

**Keywords:** inhibitor, honeycomb waste, 304 stainless steel, corrosion, electrochemical

## Abstract

The extract of honeycomb waste was studied as a corrosion inhibitor on 304 stainless steel in H_2_SO_4_ solutions. The honeycomb waste was obtained from beekeeping at Lawang-Malang, East Java, Indonesia. Electrochemical and scanning electron microscopy methods were used to investigate the performance of the corrosion inhibition process. The inhibition efficiency of the inhibitor (2000 mg/L) reached 97.29% in 0.5 M H_2_SO_4_ and decreased with the acid concentration. Kinetic parameters were calculated to explain the effect of acid concentration on the inhibition process. The study on the adsorption behavior of the extracts followed the Frumkin isotherm model. The adsorption of the inhibitor on the 304 stainless steel surface was confirmed by the negative and lower values of Gibbs free energy. The obtained scanning electron microscopy (SEM) images were confirmed by comparing the surface of the specimens with and without inhibitor after corroding for one week. The results indicated that the extract acted as a good inhibitor for 304 stainless steel in acid corrosion.

## 1. Introduction

Honey industries generally extract honey by squeezing the honeycomb; at this point, the squeezed honeycomb is damaged and becomes an organic waste that has no market value. This honeycomb waste contains bees wax and honey sugars (i.e., fructose, glucose and sucrose). It has a refractive index of 1.4398–1.4451 at 75 °C, a melting point of 61–65 °C, an acid number of 17–22, an ester number of 70–90, and a saponication number of 87–102. Water and hydrocarbon content are less than 1% and lower than 14.5%, respectively [1]. However, the honeycomb waste still contains relatively high flavonoids since a small amount of the honey remains after the squeezing process [2]. It is reported that the organic flavonoids structures have an electronegative atom, conjugated double bonds or aromatic rings that can be exploited as a corrosion inhibitor [3,4,5,6]. Therefore, in this work, we evaluated the potential of honeycomb waste as a corrosion inhibitor for structural steel materials.

Because of its formability and corrosion resistance, 304 SS is one of the materials which is widely applied in industries [7,8,9]. The corrosion resistance property is due to the protective film of chromium oxyhydroxide [10]. However, particularly in acid solutions, 304 SS is very susceptible to corrosion due to the breakage of the passive film. The most effective and economical measure to overcome this problem is the employment of an inhibitor [9,11,12,13,14]. In the present work, we investigated the performance of the honeycomb waste extract as a corrosion inhibitor in various concentrations of H_2_SO_4_ solution. The performance of the inhibitor was evaluated using potentiodynamic polarization, electrochemical impedance spectroscopy (EIS) and scanning electron microscopy (SEM) methods.

## 2. Materials and Methods 

### 2.1. Materials

The experiments were conducted on 304 SS (0.04% C, 0.52 % Si, 0.92% Mn, 0.030% P, 0.002% S, 9.58% Ni, 18.15% Cr and the balance Fe, in wt.). The dimension of the specimen was 4 cm × 1 cm × 1 mm. All parts of the specimen were covered by epoxy resin, except for 1 cm^2^ on each end of the specimen. The uncovered part was abraded using emery paper with a grade of 500 and 2000 consecutively. Furthermore, they were washed thoroughly with demineralized water and acetone.

The acid solutions were prepared by diluting AR grade 98% H_2_SO_4_ (Merck, Darmstadt Germany). Honeycomb waste was obtained from Rimba Raya beekeeping at Lawang-Malang, East Java, Indonesia. Fifty grams of honeycomb powder was extracted by the maceration method using 50 mL of 99% ethanol (Merck). This mixture was shaken for 3 h then transferred into a separating funnel. The organic extract from bees wax propolis was obtained by evaporating the bees wax propolis waste.

### 2.2. Characterization of Honeycomb Waste Extract

The extract was characterized by Fourier-transform infrared spectroscopy (FT-IR; Shimadzu IR Prestige-21, Kyoto, Japan) using the KBr-disk technique. The extract was also analyzed using an ultra-high-performance liquid chromatography (UHPLC)-ACCELLA 1250 System (Thermo Scientific, Pasadena, CA, USA). The separation was performed using a Hypersil Gold analytical column, Pasadena, CA, USA (50 mm × 2.1 mm, 1.9 µm particle size) at 30 °C with a flowrate of 300 µL/min. Gradient elution was carried out with a binary system consisting of (A) 0.1% formic acid in bi-distilled water and (B) 0.1% formic acid in acetonitrile. The gradient elution setting was adjusted as follows: 0–0.6 min, 15% B; 2–4 min 100% B; 4 min 15% B; 6 min 20% B; 10 min 45%; 20 min 25% B. A 2 µL of sample was injected for analysis.

The tandem mass spectrometry (MS/MS) triple quadrupole mass spectrometer (TSQ QUANTUM ACCESS MAX from Thermo Finnigan, Stanford, UK) with an electrospray ionization source (ESI) operating in negative ionization mode equipped with TSQ Tune software (Stanford, UK) was used for compound identification. The operational conditions of ESI ionization were as follows: spray voltage, 3 kV; evaporation temperature, 250 °C; capillary temperature, 300 °C; pressure of sheath gas, 40 psi; and aux gas pressure, 10 psi. The relative amount of each compound in the extracts was also calculated. Identification of the compounds was conducted by evaluating the chromatogram and mass spectra with the standard library.

### 2.3. Electrochemical Measurement

Electrochemical experiments were carried out using three-electrode-cell system with platinum as the counter electrode (CE), Ag/AgCl (3 M KCl) as the reference electrode (RE) and the 304 SS specimen as the working electrode (WE). The test solution was prepared by dissolving 0, 1000, 2000, 3000 and 4000 mg/L inhibitor in each acid solution, that is, 0.5, 1.0, 1.5 and 2.0 M H_2_SO_4_ solution. All tests were carried out at 25 °C.

The electrode was immersed in the test solution at open-circuit potential (OCP) to stable conditions before measurement. All experiments were carried out using Autolab PGSTAT128N (Herisau, Switzerland) equipped with Nova 1.11 software. The potentiodynamic polarization was measured at ±200 mV from *E*_corr_ with a scan rate of 1 mV/s [15]. The Tafel plots obtained were extrapolated to get the corrosion parameters. Inhibition efficiency (*IE* %) was defined as:

(1) $$IE\;\left(\%\right)=\frac{I_{\mathrm{corr}}-I_{\mathrm{corr}\left(i\right)}}{I_{\mathrm{corr}}}\times 100$$

where *I*_corr_ and *I*_corr(*i*)_ represented the corrosion current density with and without inhibitor, respectively.

Electrochemical impedance spectroscopy (EIS) was performed at open-circuit potential (OCP) in the frequency range of 1000 to 0.1 Hz using the signal amplitude of 15 mA. *IE*% from the EIS method was calculated using the following equation (Equation (2)):

(2) $$IE\;\left(\%\right)=\frac{R_{\mathrm{ct}\left(i\right)}-R_{\mathrm{ct}}}{R_{\mathrm{ct}\left(i\right)}}\times 100$$

where *R*_ct_ and *R*_ct(*i*)_ were the charge transfer resistance with and without inhibitor, respectively.

### 2.4. Surface Analysis

The specimens were immersed in 1.0 M, 1.5 M and 2.0 M H_2_SO_4_ solution with and without inhibitor (2000 mg/L) for one week at ambient temperature. Then, the specimens were washed with demineralized water and dried at room temperature. The morphological structure of the 304 SS surface was observed using a scanning electron microscope (SEM; FEI Inspect S-50, Tokyo, Japan). Before observation, all specimens were sputtered with a 10 nm layer of gold.

## 3. Results

### 3.1. Characterization of Honeycomb Waste Extract

**Figure 1:**
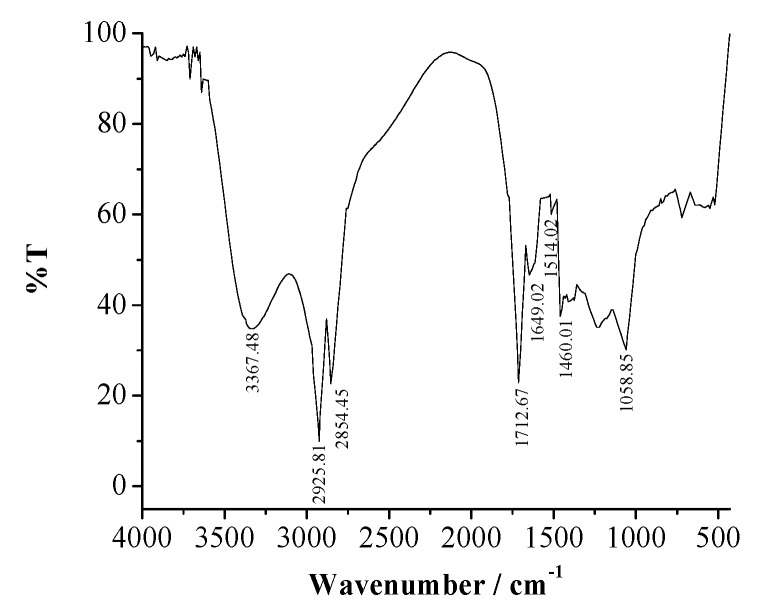
Fourier-Transform Infrared (FTIR) spectra of honeycomb waste extract.

Figure 1 shows the characteristic of the flavonoid wavenumber. It includes a carbonyl group (C=O, ketone) at λ = 1712.67 cm^−1^, O–H group at λ = 3367.48 cm^−1^ and aromatic group (C=C–C=C) at λ = 1649.02; 1514.02; and 1460.01 cm^−1^.

**Figure 2:**
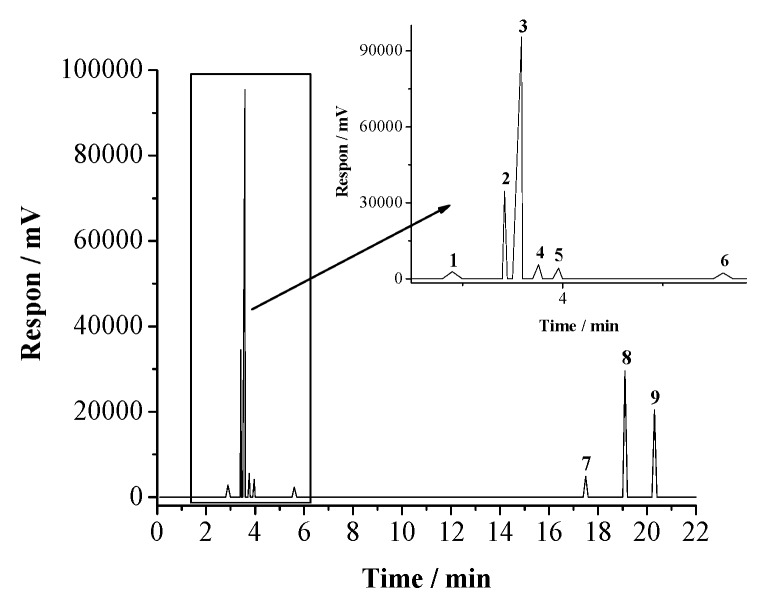
Chromatogram of honeycomb waste extract.

**Figure 3:**
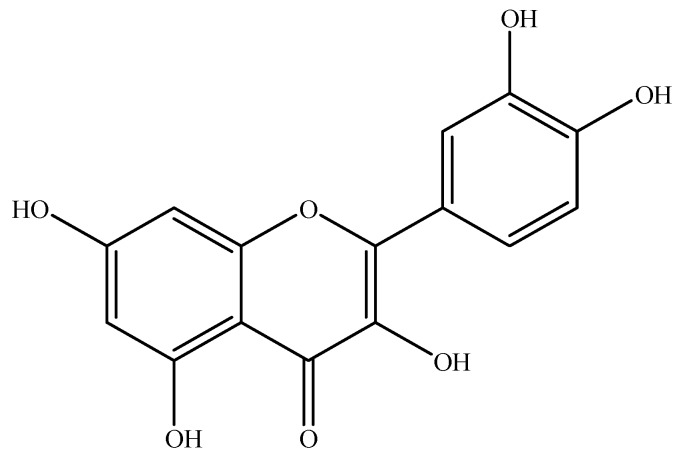
Molecule structure of quercetin.

Chromatograph of the extract displays nine peaks, which indicates nine different compounds (Figure 2). A major compound is found at a retention time of 3.59 min (peak 3) with an area of 43.90%, which is identified as quercetin (Figure 3). Identification of the other peaks gives the following results: luteolin, 1; vitexin, 2; fisetin, 4; isohamnetin, 5; isoferulic, 6; apigenin, 7; pinobanksin, 8; and kaempferol, 9. The detailed analysis of these compounds is summarized in Table 1. 

The active sites of the main compound in the extract can interact with the vacant d orbital of Fe and form a thin protective layer [16,17]. The presence of an inhibitor gives two specific adsorbed intermediates to determine the anodic dissolution of Fe by the following mechanism:

(3a) $$\mathrm{Fe}+H_{2}O↔{\mathrm{FeH}}_{2}O_{\mathrm{ads}}$$

(3b) $${\mathrm{FeH}}_{2}O_{\mathrm{ads}}+M↔{\mathrm{FeOH}}_{\mathrm{ads}}^{-}+H^{+}+M$$

(3c) $${\mathrm{FeH}}_{2}O_{\mathrm{ads}}+M↔{\mathrm{FeM}}_{\mathrm{ads}}+H_{2}O$$

(3d) $${\mathrm{FeOH}}_{\mathrm{ads}}^{-}\overset{\mathrm{rds}}{\to }{\mathrm{FeOH}}_{\mathrm{ads}}+e$$

(3e) $${\mathrm{FeM}}_{\mathrm{ads}}↔{\mathrm{FeM}}_{\mathrm{ads}}^{+}+e$$

(3f) $${\mathrm{FeOH}}_{\mathrm{ads}}+{\mathrm{FeM}}_{\mathrm{ads}}^{+}↔{\mathrm{FeM}}_{\mathrm{ads}}+{\mathrm{FeOH}}^{+}$$

(3g) $${\mathrm{FeOH}}^{+}+H^{+}↔{\mathrm{Fe}}^{2+}H_{2}O$$

where M represents the inhibitor species. 

There are some active sites on the surface of corroded metal that are able to absorb activation energy. In this case, the inhibitors molecule can be adsorbed easily on the active sites of the surface with matched adsorption enthalpies. According to Equation (3a–g), water molecules on the metal surface are replaced to yield the adsorption of intermediate FeM_ads_ by inhibitor species (Equation (3e)). This reduces the amount of the species FeOH_ads_ which causes retardation of Fe anodic dissolution [16,18,19].

### 3.2. Potentiodynamic Polarization

**Figure 4:**
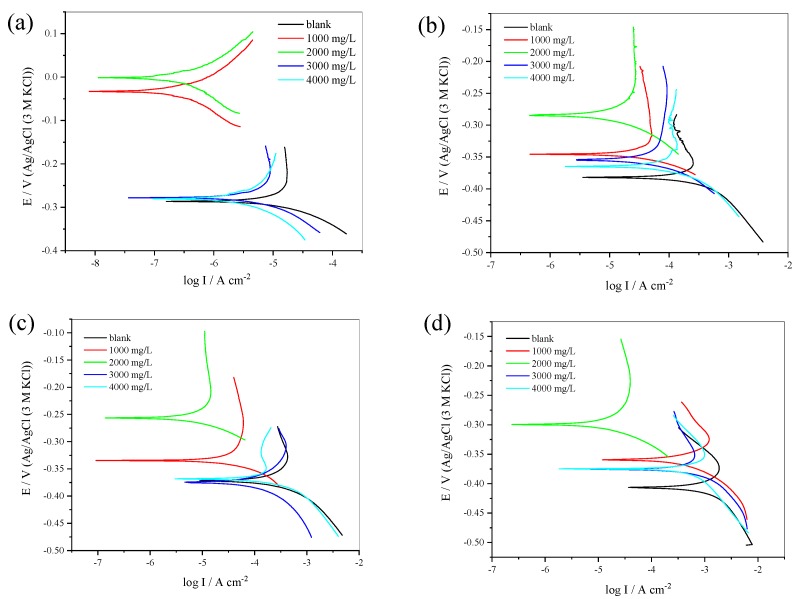
Tafel plots for 304 SS with and without inhibitor in 0.5 M (**a**), 1.0 M (**b**), 1.5 M (**c**), and 2.0 M H_2_SO_4_ solution (**d**).

The *E*_corr_ of 304 SS shifts to a higher potential with a similar pattern in all acid concentrations in comparison with the blank. The 2000 mg/L inhibitor gives the maximum shift difference (Figure 4) with the shift to the blank, which are 28.5 mV, 98.0 mV, 115.0 mV and 104 mV in 0.5 M, 1.0 M, 1.5 M and 2.0 M H_2_SO_4_ solutions, respectively. It confirms that the inhibitor acts as an anodic inhibitor in 1.0 M, 1.5 M and 2.0 M H_2_SO_4_ solutions (the displacement of the *E*_corr_ is more than 85.0 mV) but in 0.5 M the inhibitor acts as a mixed inhibitor [20]. This is also confirmed by the value of the anodic current density, which is lower than the cathodic.

The maximum concentration that gives the highest number in *IE*% is 2000 mg/L (Table 2). The corrosion rate of 304 SS decreases with an inhibitor of 1000–2000 mg/L and increases with a 3000–4000 mg/L inhibitor. It can be attributed to the adsorption behavior of the inhibitor on 304 SS/acid solution interface [9,21]. The increase of inhibitor concentration beyond 2000 mg/L leads to diminished corrosion protection. This may be caused by the withdrawal of inhibitor back into the bulk solution when the inhibitor concentration is close to or beyond the critical concentration. This leads to a weakening of the metal-inhibitor interactions and causes the replacement of the inhibitor with water or SO_4_^2−^, reducing its *IE*% [21]. 

### 3.3. Electrochemical Impedance Spectroscopy (EIS) Measurement 

**Figure 5:**
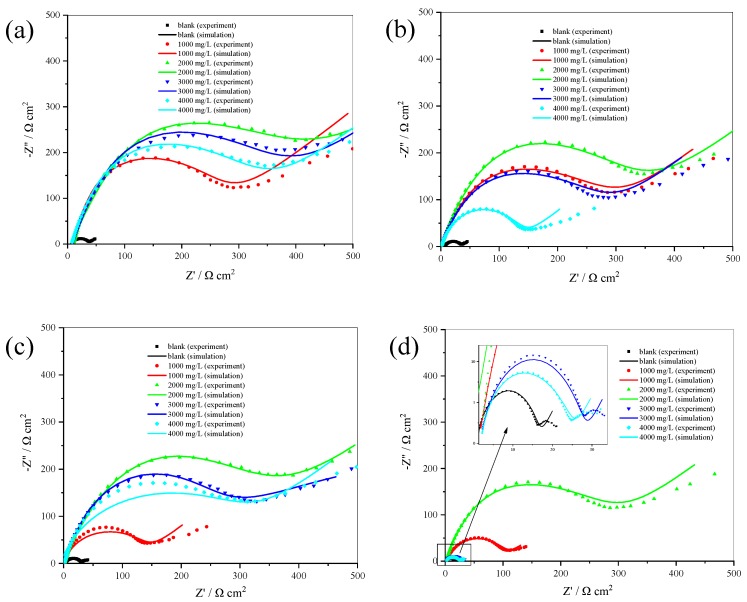
Nyquist plots for 304 SS with and without inhibitor in 0.5 M (**a**), 1.0 M (**b**), 1.5 M (**c**), and 2.0 M H_2_SO_4_ solution (**d**).

The performance of honeycomb waste extract as an inhibitor at corroding 304 SS in sulfuric acid was also studied by electrochemical impedance spectroscopy (EIS). Figure 5 shows the Nyquist plots of 304 SS with and without inhibitor. According to Figure 5, there is a semicircle at high frequencies and a straight line at low frequencies. High frequency semicircles are generally associated with the charge transfer at the electrode/electrolyte interface, such as an electrical double layer. A straight line at low frequencies indicate Warburg impedance (*W*) [22]. *W* attributed to the anodic diffusion process of oxygen transport from the bulk solution to the electrode surface [23].

**Figure 6:**
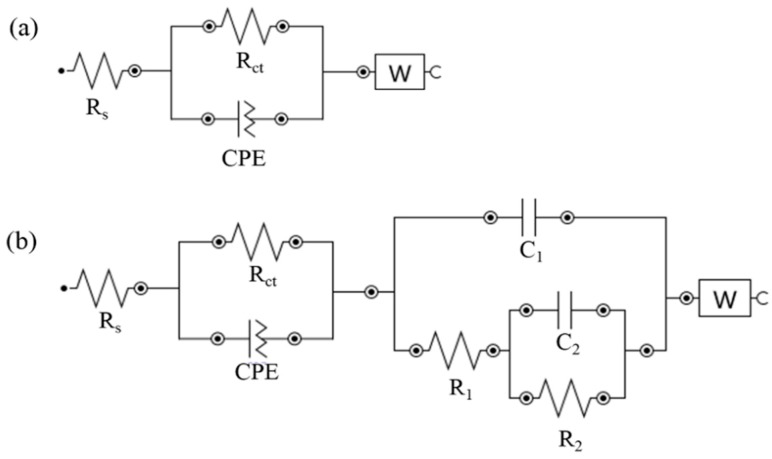
Equivalent circuit used to the EIS spectra for the absence (**a**) and the presence (**b**) of inhibitor in 304 SS corrosion at sulfuric acid solution.

Figure 6a,b are equivalent circuits used for the EIS spectra for 304 SS in both the absence and the presence of inhibitor, respectively. Figure 6a is the standard equivalent circuit with 4 parameters, that is, *R*_s_, *R*_ct_, CPE and *W*. *R*_s_ represents the solution resistance, *R*_ct_ is correlated with the charge transfer resistance of metal, CPE is the constant phase element and *W* is the Warburg impedance. A more complicated equivalent circuit occurred with the addition of the inhibitor (Figure 6b). According to Figure 6b, there are two parts of equivalent circuit when the first part (*R*_s_, *R*_ct_ and CPE) is the standard and the second part (*R*_1_, *R*_2_, *C*_1_ and *C*_2_) is the additional circuit. The first part indicates that the inhibitor attaches to the metal surface, which is shown by the increasing in the value of *R*_ct_. The *R*_ct_ increases along with the increased inhibitor concentration which is up to 2000 mg/L; a further increase of the concentration leads to a decrease in the value of *R*_ct_ (Table 3). The *R*_ct_ is inversely proportional to the corrosion rate and related to *IE*% [21,23]. The higher *R*_ct_, the lower the 304 SS corrosion rate. Therefore, the inhibition process is more efficient. It is in line with the result of potentiodynamic polarization. Moreover, additional parameters appeared in the second part of the equivalent circuit that give more information about the effect of the inhibitor. These parameters are related to the formation of the other passive films on the metal surface [24]. It is probably due to the reaction between the inhibitor and the metal surface. The fitting results for electrochemical impedance spectroscopy (EIS) data for 304 SS in several H_2_SO_4_ concentration with and without inhibitor was summarized in Table 3.

**Figure 7:**
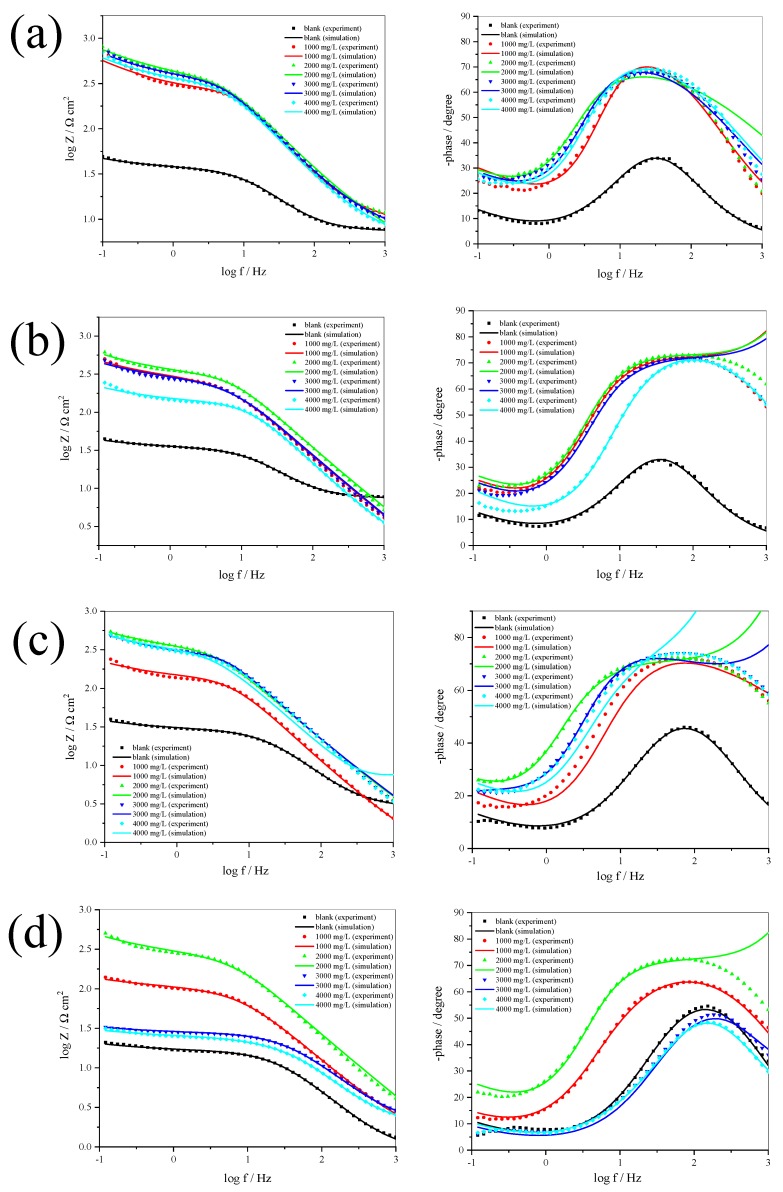
Bode plots for 304 SS with and without inhibitor in 0.5 M (**a**), 1.0 M (**b**), 1.5 M (**c**), and 2.0 M H_2_SO_4_ solution (**d**). Bode modulus (left) and Bode phase (right).

The Bode plots consist of a one loop capacitive (Figure 7a–d). It indicates that the inhibitor is adsorbed on the 304 SS surface by the gradual replacement of water molecules and ions. Figure 7 shows that increasing inhibitor concentration up to 2000 mg/L results in a more negative value of the phase angle. It showed that there is greater surface coverage and transfer charge resistance [25,26,27].

### 3.4. The Effect of Acid Concentration

**Figure 8:**
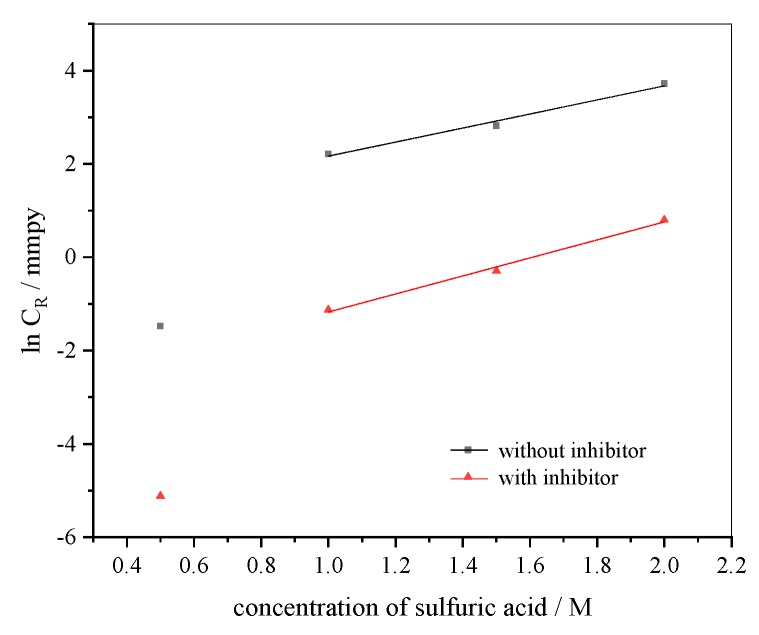
The straight lines of lnC_R_ versus acid concentration with and without inhibitor at 25 °C.

The relationship of the corrosion rate (*C*_R_) against acid concentration (*C*) obeys the kinetic equation:

(4) $$\ln C_{R}=\ln k+BC$$

where *k* is the rate constant and *B* is the reaction constant. The relationship ln*C*_R_ versus *C* gives straight lines as shown in Figure 8. The slopes and intercept of these lines represent the *B* constant and ln*k*, respectively.

*k* can be deemed a commencing rate at zero acid concentration. Therefore, *k* means the corrosion ability of acid for metal. *B* represents the difference of the corrosion rate at the acid concentration. The decreased k with inhibitor (Table 4) means that the extracts inhibit the corrosion process of 304 SS [28]. The decreased *B* with inhibitor (Table 4) indicates that the changes of the corrosion rate in inhibited acid are larger than in uninhibited acid [28].

### 3.5. Adsorption Isotherm and Thermodynamic Calculations

**Figure 9:**
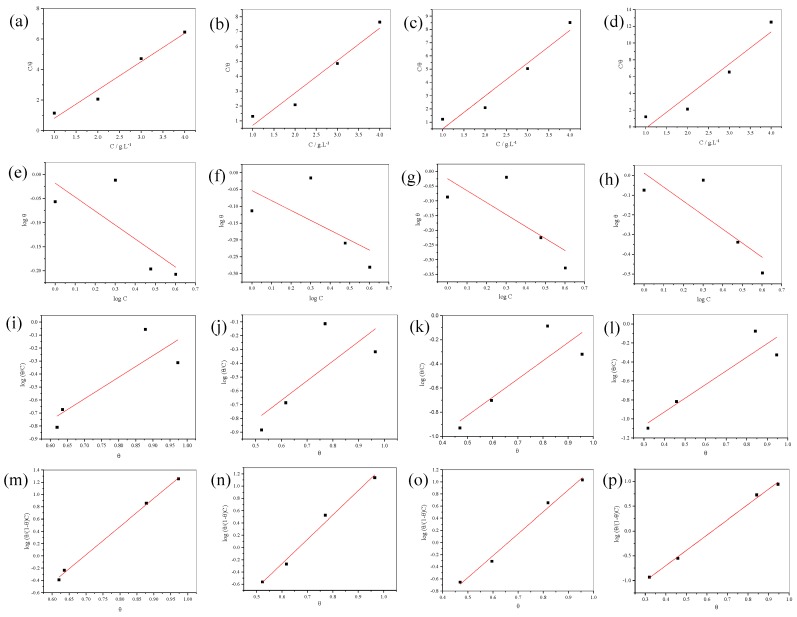
Langmuir (**a**–**d**), Freundlich (**e**–**h**), Temkin (**i**–**l**) and Frumkin isotherm (**m**–**p**) for the adsorption of the inhibitor on 304 SS surface in 0.5 M, 1.0 M, 1.5 M and 2.0 M H_2_SO_4_, respectively.

The corresponding plots of the adsorption isotherm are shown in Figure 9. These are the Langmuir (Figure 9a–d), Freundlich (Figure 9e–h), Temkin (Figure 9i–l) and Frumkin isotherms (Figure 9m–p). The best fit is shown by *R*–square (Table 5), for this study follows the Frumkin isotherm (Equation (5)).

Frumkin equation:

(5) $$\log\left\{\frac{\theta }{\left(1-\theta \right)C}\right\}=\log K_{\mathrm{ads}}+a\theta$$

where *C* is the inhibitor concentration, θ is the surface coverage, *K*_ads_ is the adsorption equilibrium constant and *a* is an interaction parameter. The value of *a* indicates attraction or repulsion between adsorbed species for *a* > 0 and *a* < 0, respectively. When *a* = 0, it means no interaction and it becomes equivalent to the Langmuir isotherm [29]. The Frumkin isotherm considers lateral interactions between adsorbed inhibitor molecules indicating that the inhibitor displaces the water molecules from the 304 SS surface [30]. The interaction parameters were calculated from the slope of Figure 9m–p. The positive value of a (Table 6) indicates highly attractive lateral interactions in the adsorbed layer. The increase of the inhibitor concentration probably induces desorption of the inhibitor, which is already adsorbed at the 304 SS surface and then it dissolves into solution. It makes the interactions stronger between the inhibitor in the solution and leads to secondary desorption [29,30].

*K*_ads_ is related to the Gibbs free energy of adsorption ($\Delta G_{\mathrm{ads}}^{{}^{\circ}}$) by the Equation (6) [14].

(6) $$\Delta G_{\mathrm{ads}}^{{}^{\circ}}=-RT\ln\left(K_{\mathrm{ads}}\times A\right)$$

where *A* is the concentration of water (55.5 in M or 1000 in g/L), *T* is the absolute temperature and *R* is the universal gas constant.

The thermodynamic parameters are summarized in Table 6. The negative and lower values of $\Delta G_{\mathrm{ads}}^{{}^{\circ}}$ indicate the inhibition process of the inhibitor on the 304 SS surface, which is spontaneous, and physisorption [13,31]. The more negative value follows the order 0.5 M H_2_SO_4_ > 1.0 M H_2_SO_4_ > 1.5 M H_2_SO_4_ > 2.0 M H_2_SO_4_.

The heat of adsorption ($\Delta H_{\mathrm{ads}}^{{}^{\circ}}$) is calculated using the Van’t Hoff equation:

(7) $$\ln K_{\mathrm{ads}}=\frac{-\Delta H_{\mathrm{ads}}^{{}^{\circ}}}{RT}$$

The entropy of adsorption ($\Delta S_{\mathrm{ads}}^{{}^{\circ}}$) can be obtained using Equation (8).

(8) $$\Delta G_{\mathrm{ads}}=\Delta H_{\mathrm{ads}}^{{}^{\circ}}-T\Delta S_{\mathrm{ads}}^{{}^{\circ}}$$

The negative value of $\Delta H_{\mathrm{ads}}^{{}^{\circ}}$ confirms the exothermic nature of the metal dissolution process with the inhibitor [31]. The positive value of $\Delta S_{\mathrm{ads}}^{{}^{\circ}}$ means the adsorption process is accompanied by an increase in entropy [18].

### 3.6. Surface Analysis

**Figure 10:**
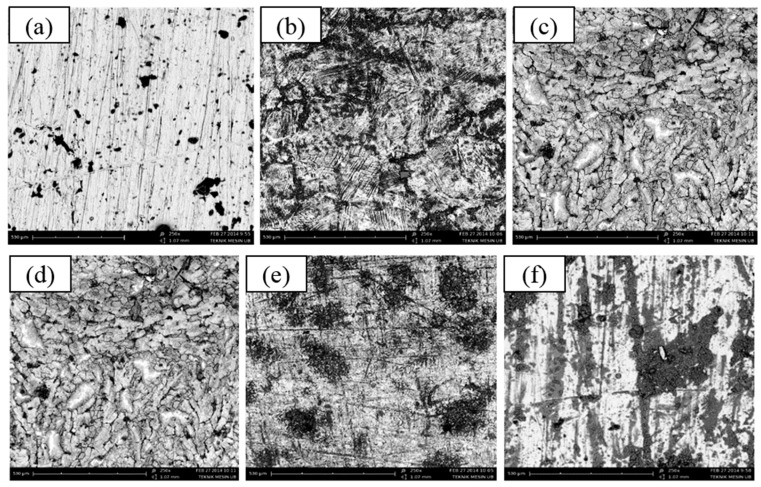
Scanning electron spectroscopy (SEM) images of the 304 SS surface in 250× before corrosion (**a**), after corrosion in 1.0 M H_2_SO_4_ without inhibitor (**b**) after corrosion in 1.0 M H_2_SO_4_ with inhibitor 1000 mg/L (**c**), after corrosion in 1.0 M H_2_SO_4_ with inhibitor 2000 mg/L (**d**), after corrosion in 1.0 M H_2_SO_4_ with inhibitor 3000 mg/L (**e**), after corrosion in 1.0 M H_2_SO_4_ with inhibitor 4000 mg/L (**f**).

In order to provide physical evidence, SEM analysis was conducted. The impact of the inhibitor on the microstructure of the top surface of 304 SS is depicted in Figure 10. The surface of 304 SS is relatively smooth before the corrosion process (Figure 10a). After being corroded in acid solutions in the absence of the inhibitor, some cracks and pits appear on the 304 SS surface (Figure 10b); meanwhile, in the presence of the inhibitor, there is less damage (Figure 10c–f). It shows that the inhibitor works well at protecting against corrosion.

## 4. Conclusions

The honeycomb waste extracts can be used as an effective inhibitor for 304 SS corrosion in H_2_SO_4_ solutions. The FTIR spectrum indicates that the extract has several functional groups which are typical for a flavonoid compound. Furthermore, LC–MS analysis shows that the main compound of the extract was quercetin. The *IE*% maximum in this study was obtained by adding 2000 mg/L inhibitors in 0.5 M H_2_SO_4_, that is, 97.29% using potentiodynamic polarization. The extracts adsorption behavior of the extracts follow the Frumkin isotherm model. The corrosion inhibition performance of the extracts on the surface of 304 SS decreases with acid concentration.

## Figures and Tables

**Table 1:** Identification of the honeycomb waste extracts using liquid chromatography-mass spectrometry (LC-MS).

Peak	RT (min)	Area (uV s)	Height (uV)	Area (%)	Molecular Weight	Molecular Formula	Compound
1	2.90	16,787.68	2813.08	1.17	286.24	C_15_H_10_O_6_	Luteolin
2	3.42	214,035.89	34,555.55	14.96	432.00	C_21_H_20_O_10_	Vitexin
3	3.59	628,240.32	95,418.53	43.90	301.90	C_15_H_10_O_7_	Quercetin
4	3.76	35,082.10	5569.59	2.45	287.00	C_15_H_10_O_6_	Fisetin
5	3.96	25,177.60	4213.24	1.76	315.00	C_16_H_12_O_7_	Isohamnetin
6	5.60	13,670.09	2334.22	0.96	194.00	C_10_H_10_O_4_	Isoferulic
7	17.50	39,787.95	4860.57	2.78	270.24	C_15_H_10_O_5_	Apigenin
8	19.1	241,347.00	29,633.18	16.87	272.00	C_15_H_12_O_5_	Pinobanksin
9	20.3	216,906.26	20,458.26	15.16	286.00	C_15_H_10_O_6_	Kaempferol

**Table 2:** Corrosion parameters for 304 SS in several H_2_SO_4_ concentrations with and without inhibitor.

[H_2_SO_4_]	*C*_inh_ (mg/L)	βa (V/dec)	βc (V/dec)	*E*_corr_ (V)	*I*_corr_ (A/cm^2^)	*C*_R_ (mmpy)	*IE* (%)
0.5 M	0	0.059	−7.958	−0.286	2.19 × 10^−5^	0.228	–
	1000	0.224	−0.794	−0.033	2.67 × 10^−6^	0.028	87.79
	2000	0.103	0.172	−0.001	5.93 × 10^−7^	0.006	97.29
	3000	0.062	0.359	−0.278	7.97 × 10^−6^	0.083	63.60
	4000	0.082	−0.707	−0.280	8.32 × 10^−6^	0.087	61.99
1.0 M	0	0.073	−0.422	−0.382	8.83 × 10^−4^	9.186	–
	1000	0.056	−0.111	−0.346	2.03 × 10^−4^	2.116	76.96
	2000	0.075	0.442	−0.284	3.10 × 10^−5^	0.323	96.49
	3000	0.072	−0.138	−0.354	3.38 × 10^−4^	3.513	61.75
	4000	0.063	−0.168	−0.365	4.21 × 10^−4^	4.376	52.36
1.5 M	0	0.101	−0.238	−0.372	1.62 × 10^−3^	16.793	–
	1000	0.074	−0.119	−0.335	2.94 × 10^−4^	3.053	81.82
	2000	0.098	−0.181	−0.257	7.17 × 10^−5^	0.745	95.56
	3000	0.053	−0.103	−0.362	6.55 × 10^−4^	6.807	59.46
	4000	0.087	−0.166	−0.364	8.59 × 10^−4^	8.929	46.82
2.0 M	0	0.165	0.360	−0.403	3.97 × 10^−3^	41.311	–
	1000	0.066	0.069	−0.359	6.26 × 10^−4^	6.509	84.24
	2000	0.118	−0.189	−0.299	2.14 × 10^−4^	2.227	94.61
	3000	0.068	−0.345	−0.376	2.15 × 10^−3^	22.325	45.96
	4000	0.649	0.129	−0.375	2.70 × 10^−3^	28.086	32.01

**Table 3:** Fitting results of electrochemical impedance spectroscopy (EIS) data for 304 SS in several H_2_SO_4_ concentration with and without inhibitor.

[H_2_SO_4_]	*C*_inh_ (mg/L)	*R*_s_ (Ω cm^2^)	*R*_ct_ (Ω cm^2^)	CPE (F/cm^2^)	*n*	*R*_1_ (kΩ cm^2^)	*R*_2_ (kΩ cm^2^)	*C*_1_ (F)	*C*_2_ (F)	*W Y*_W_ (Ω^−1^ cm^−2^ s*^n^*)	χ^2^
0.5 M	0	7.307	27.961	8.30 × 10^−4^	0.8599	–	–	–	–	0.0839	0.0378
	1000	7.584	252.100	9.91 × 10^−5^	1.0099	−3.896	3.846	3.70 × 10^−4^	9.00 × 10^−13^	0.0032	0.2389
	2000	2.797	389.97	1.80 × 10^−4^	0.8855	−33.827	33.744	4.10 × 10^−4^	9.00 × 10^−13^	0.0026	0.1319
	3000	5.406	350.37	1.40 × 10^−4^	0.9418	−2.036	1.966	4.30 × 10^−4^	9.00 × 10^−13^	0.0029	0.1011
	4000	4.511	315.58	1.40 × 10^−4^	0.9560	−6.998	9.00 × 10^−16^	4.40 × 10^−4^	9.00 × 10^−13^	0.0032	0.1058
1.0 M	0	7.342	25.654	7.60 × 10^−4^	0.8674	–	–	–	–	0.0915	0.0163
	1000	12.339	227.180	1.90 × 10^−4^	0.8939	−14.090	−40.123	9.00 × 10^−13^	5.50 × 10^−4^	0.0045	0.6122
	2000	−2.207	340.760	1.30 × 10^−4^	0.9109	−70.889	2.082	3.60 × 10^−4^	8.70 × 10^−8^	0.0034	0.4138
	3000	−1.421	271.340	1.80 × 10^−4^	0.8847	−82.736	45.708	5.60 × 10^−4^	9.00 × 10^−13^	0.0049	0.6476
	4000	1.064	140.780	1.50 × 10^−4^	0.9336	−59.671	43.807	8.70 × 10^−4^	9.00 × 10^−13^	0.0115	0.1446
1.5 M	0	2.779	25.607	5.30 × 10^−4^	0.862	–	–	–	–	0.1009	0.0279
	1000	−34.995	122.820	2.60 × 10^−4^	0.949	−41.686	76.859	9.00 × 10^−13^	9.00 × 10^−13^	0.0113	0.3348
	2000	−2.944	342.560	2.60 × 10^−4^	0.895	−4616.100	4547.000	8.90 × 10^−4^	9.00 × 10^−13^	0.0039	0.0438
	3000	−395.090	264.590	1.60 × 10^−4^	0.954	164.050	230.330	9.00 × 10^−13^	9.00 × 10^−13^	0.0044	0.7933
	4000	−85.645	257.450	2.30 × 10^−4^	0.915	−4308.000	4385.300	9.00 × 10^−13^	8.35 × 10^−12^	0.0433	0.6357
2.0 M	0	0.865	15.330	0.87 × 10^−2^	0.866	–	–	–	–	0.2329	0.0513
	1000	1.173	105.700	4.00 × 10^−4^	0.845	−97.542	90.361	3.11 × 10^−3^	6.23 × 10^−7^	0.0284	0.0042
	2000	−1.762	276.930	1.90 × 10^−5^	0.895	−15.848	−24.151	5.50 × 10^−4^	5.54 × 10^−10^	0.0045	0.6122
	3000	−52.314	25.708	4.10 × 10^−4^	0.839	35.063	19.000	1.44 × 10^−8^	9.00 × 10^−13^	0.1715	0.0533
	4000	3.809	17.723	1.15 × 10^−3^	0.777	−2.083	5.033	3.40 × 10^−6^	6.00 × 10^−4^	0.1701	0.0279

**Table 4:** Calculation of the linear regression between ln*C*_R_ and *C* for the 304 SS corrosion in H_2_SO_4_ solution.

Systems	*R* ^2^	*k* (mmpy)	*B* (M^−1^)
Blank	0.987	1.944	1.503
Inhibitor	0.994	0.045	1.931

**Table 5:** *R*–Square of various isotherm for the adsorption of the inhibitor on 304 SS surface in 0.5 M, 1.0 M, 1.5 M and 2.0 M H_2_SO_4_, respectively.

Systems	Isotherm	*R* ^2^
in 0.5 H_2_SO_4_	Langmuir	0.9706
	Freundlich	0.5882
	Temkin	0.7285
	Frumkin	0.9979
in 1.0 M H_2_SO_4_	Langmuir	0.9515
	Freundlich	0.4410
	Temkin	0.6193
	Frumkin	0.9884
in 1.5 M H_2_SO_4_	Langmuir	0.9443
	Freundlich	0.5890
	Temkin	0.7574
	Frumkin	0.9922
in 2.0 M H_2_SO_4_	Langmuir	0.9186
	Freundlich	0.6936
	Temkin	0.8691
	Frumkin	0.9973

**Table 6:** The thermodynamic parameters for inhibitor adsorption on the 304 SS surface at ambient temperature (25 °C).

Systems	*a*	|log *K*_ads_|	$\Delta G_{\mathrm{ads}}^{{}^{\circ}}$ (kJ/mol)	$\Delta H_{\mathrm{ads}}^{{}^{\circ}}$ (kJ/mol)	$\Delta S_{\mathrm{ads}}^{{}^{\circ}}$ (J/mol K)
in 0.5 H_2_SO_4_	4.586	3.191	−35.301	−18.195	57.40
in 1 M H_2_SO_4_	3.976	2.650	−32.216	−15.110	57.40
in 1.5 M H_2_SO_4_	3.613	2.383	−30.694	−13.588	57.40
in 2 M H_2_SO_4_	3.086	1.932	−28.123	−11.016	57.41
